# An infected false aneurysm of the subclavian artery in a 41-year old drug abuser

**DOI:** 10.11604/pamj.2015.22.211.7801

**Published:** 2015-11-05

**Authors:** Melek Ben Mrad, Bilel Derbel

**Affiliations:** 1 Departement of Cardiovascular Surgery, La Rabta Hospital, Faculty of Medecine of Tunis, University Tunis EL Manar, Tunisia

**Keywords:** Drug abuse, pseudo-aneurysm, subclavian artery

## Image in medicine

A 41-year-old man, with a history of intravenous drug abuse, presented with a mass in the right side of the neck. The mass had increased in size over a three-day period, producing local pain, hoarseness, shortness of breath, fever and pain of the right arm. Anamnesis told he performed internal jugular drug injection on a regular base. Physical examination revealed a pulsatile supraclavicular mass, with central necrotic lesions (A). Chest X-ray showed an opacity of the right hemithorax with left tracheal deviation (B). Additional computed tomographic angiography revealed a false aneurysm of the right subclavian artery, resulting in a filling defect of the axillary artery causing malperfusion (C). A midsternotomy with lateral extension was performed, which revealed an infected hematoma of a ruptured pseudo-aneurysm of the right subclavian artery. After ligation of the feeding vessels and debridement of the false aneurysm, an iatrogenic lesion of the branchio-cephalic trunk occurred, requiring interposition graft to the common carotid artery. Subsequently, a bypass graft from brachio-cephalic trunk to the humeral artery was performed (D). Postoperative course was complicated by an acute disseminated intravascular coagulation eventually leading to death An infected pseudoaneurysm of the subclavian artery as a consequence of intravenous drug use is extremely rare, with only 7 cases reported in the literature. It's a very grave entity, the risk of death is very important.

**Figure 1:**
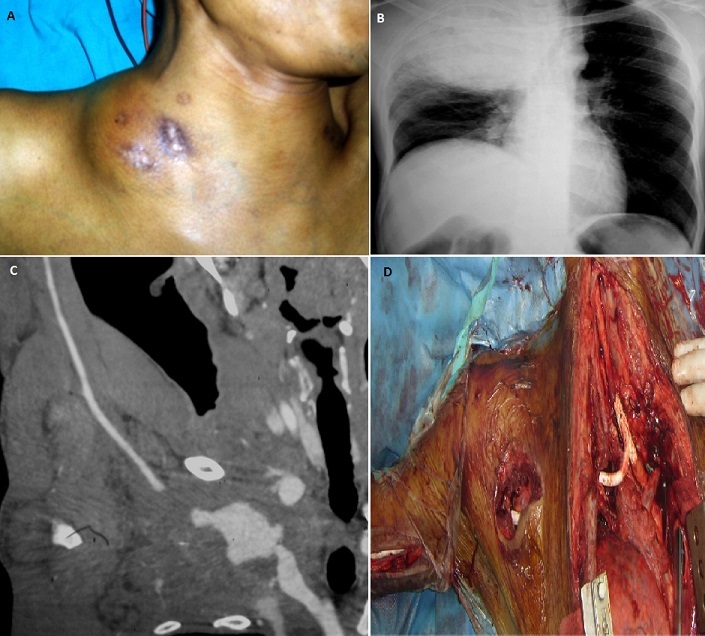
A) a supraclavicular mass, with central necrotic lesions; B) X-ray showing an opacity of the right hemithorax with left tracheal deviation; C) computed tomographic angiography showing a false aneurysm of the right subclavian artery; D) bypass graft from brachio-cephalic trunk to the humeral artery and carotid artery

